# Identification of molecular signatures associated with sleep disorder and Alzheimer’s disease

**DOI:** 10.3389/fpsyt.2022.925012

**Published:** 2022-08-04

**Authors:** Lucong Liang, Jing Yan, Xiaohua Huang, Chun Zou, Liechun Chen, Rongjie Li, Jieqiong Xie, Mika Pan, Donghua Zou, Ying Liu

**Affiliations:** ^1^Department of Neurology, The Second Affiliated Hospital of Guangxi Medical University, Nanning, China; ^2^Department of Geriatrics, The Fifth Affiliated Hospital of Guangxi Medical University, Nanning, China; ^3^Department of Neurology, The Affiliated Hospital of Youjiang Medical University for Nationalities, Baise, China; ^4^Department of Geriatrics, The First People’s Hospital of Nanning, Nanning, China

**Keywords:** Alzheimer’s disease, sleep disorder, gene expression, bioinformatics analysis, differentially expressed gene (DEG)

## Abstract

**Background:**

Alzheimer’s disease (AD) and sleep disorders are both neurodegenerative conditions characterized by impaired or absent sleep. However, potential common pathogenetic mechanisms of these diseases are not well characterized.

**Methods:**

Differentially expressed genes (DEGs) were identified using publicly available human gene expression profiles GSE5281 for AD and GSE40562 for sleep disorder. DEGs common to the two datasets were used for enrichment analysis, and we performed multi-scale embedded gene co-expression network analysis (MEGENA) for common DEGs. Fast gene set enrichment analysis (fGSEA) was used to obtain common pathways, while gene set variation analysis (GSVA) was applied to quantify those pathways. Subsequently, we extracted the common genes between module genes identified by MEGENA and genes of the common pathways, and we constructed protein-protein interaction (PPI) networks. The top 10 genes with the highest degree of connectivity were classified as hub genes. Common genes were used to perform Metascape enrichment analysis for functional enrichment. Furthermore, we quantified infiltrating immune cells in patients with AD or sleep disorder and in controls.

**Results:**

DEGs common to the two disorders were involved in the citrate cycle and the HIF-1 signaling pathway, and several common DEGs were related to signaling pathways regulating the pluripotency of stem cells, as well as 10 other pathways. Using MEGENA, we identified 29 modules and 1,498 module genes in GSE5281, and 55 modules and 1,791 module genes in GSE40562. Hub genes involved in AD and sleep disorder were ATP5A1, ATP5B, COX5A, GAPDH, NDUFA9, NDUFS3, NDUFV2, SOD1, UQCRC1, and UQCRC2. Plasmacytoid dendritic cells and T helper 17 cells had the most extensive infiltration in both AD and sleep disorder.

**Conclusion:**

AD pathology and pathways of neurodegeneration participate in processes contributing in AD and sleep disorder. Hub genes may be worth exploring as potential candidates for targeted therapy of AD and sleep disorder.

## Introduction

Neurodegenerative disease is a general designation for conditions involving progressive loss of structure or function of the nervous system, leading ultimately to neuronal death, including Alzheimer’s disease (AD) and prion disease (1). These diseases vary in their pathophysiology: some can cause memory and cognitive impairments, while others affect the ability to move, speak, and breathe (2, 3). The symptoms of AD begin to develop with mild memory impairment and evolve into cognitive impairment and dysfunction in complex daily activities (4). AD can be ultimately fatal. Cognitive deficits are ameliorated by reduction in amyloid β accumulation (5). AD is one of the most common factors of dementia and frailty (4), which occurs mostly in people over 65 years (6). The presence of amyloid β and tau proteins is a defining characteristic of AD, characterized by loss of neurons and synapses in the cerebral cortex and certain subcortical regions (7). Nearly half of all AD adults older than 60 years also report sleep disturbances (8). In addition, disturbed sleep or lack of sleep has been identified as one of the risk factors for AD (9).

The pathology of AD may overlap with the pathology of other diseases; for example, some patients with prion mutations are initially diagnosed with AD (10). Prion diseases are neurological disorders characterized by neuronal loss, spongiform degeneration, and activation of astrocytes or microglia cells. Although rare in humans, prion diseases can lead to dementia and ataxia (11). Fatal familial insomnia (FFI) is a prion disease that involves a mutation in codon 178 of the gene encoding the prion protein (PRNP) (12). The disease is characterized by loss of sleep, abnormal autonomic function, and selective atrophy, usually without spongiform changes (13). The thalamus is the main brain region affected in this sleep disorder (6), and FFI has been considered to be dominated by sleep disorder. The disease is currently incurable and has a mean course of 18 months, ultimately leading to death (14).

There is growing evidence that poor sleep accelerates the progression of neurodegenerative disorders and may play a role in their pathogenesis (15). Since sleep disorders are present in AD, we need to understand the pathogenic factors and mechanisms of AD and sleep disorder in order to design effective treatments. Although sleep disorders and AD present different pathogenic factors and clinical features, DNA damage or accumulation of misfolded proteins may be activated as a common mechanism in the pathogenesis of both conditions, ultimately leading to irreversible neuronal loss and cell death (16, 17).

In our previous studies, we also found several biomarkers may contribute to AD. CXCR4, EGFR, MAP4K4, IGF1R (18), and RBM8A (19) may serve as biomarkers for AD diagnosis. A study identified significant marker genes, which could be related to a potential AD molecular type (20). Studies have shown that REPS1 serves as a potential biomarker for AD and Vascular dementia that were both associated with Ras signaling (21). Furthermore, lncRNAs and miRNAs determining the progression of AD, including miR-34a (22) and long ncRNA MALAT1 (23). Chan has reported that high expression of PMP2, KIF5B, and ADD3 as common molecule between AD and sleep disorders that involved in Ras signaling (24). These genes can be used for the diagnosis and treatment of neurodegenerative diseases, and we need to further exploration and verification.

However, relevant mechanisms of AD and sleep disorder are not well characterized and need to be further explored. In the present study, we used bioinformatics analyses to investigate the similarities and differences in gene expression among patients with sleep disorder or AD in order to identify hub genes and intersecting pathways. The resulting insights may help guide experimental studies to elucidate pathways common to both types of disease.

## Materials and methods

### Data preprocessing

**FIGURE 1:**
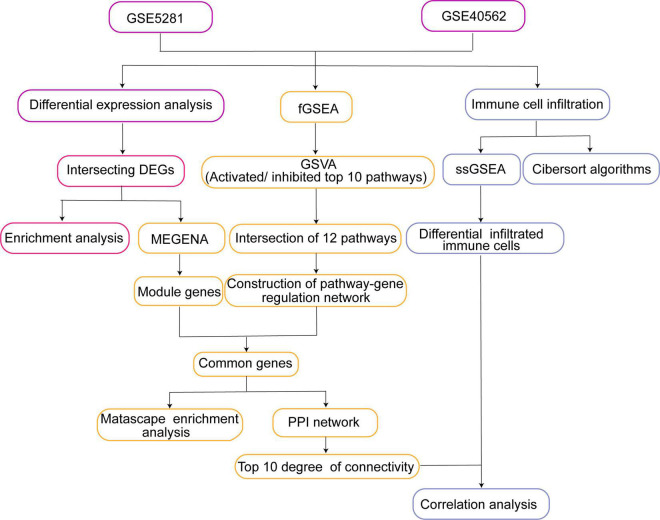
Study workflow. DEG, differentially expressed gene; fGSEA, fast gene set enrichment analysis; MEGENA, multiscale embedded gene co-expression network analysis; GSVA, gene set variation analysis; PPI, protein-protein interaction; ssGSEA, single-sample gene set enrichment analysis.

The workflow of the study is shown in Figure 1. Gene expression profiles from publicly available datasets GSE5281 and GSE40562 based on the GPL570 platform (Affymetrix Human Genome U133 Plus 2.0 Array) were downloaded from the Gene Expression Omnibus database (GEO)^1^ (25). The array data for GSE5281 consisted of 87 AD samples (obtained from six brain regions as follows: 10 entorhinal cortex, 10 hippocampus, 16 medial temporal gyrus, 9 posterior cingulate, 23 superior frontal gyrus, and 19 primary visual cortex) and 74 controls (obtained from the same six brain regions: 13 entorhinal cortex, 13 hippocampus, 12 medial temporal gyrus, 13 posterior cingulate, 11 superior frontal gyrus, and 12 primary visual cortex). The individuals included in this dataset had a mean age of 79.9 ± 6.9 years (26). A total of eight samples of thalamus and parietal lobe were analyzed in the GSE40562 dataset, from three patients with sleep disorder and one normal individual. The three patients included a 48-year-old man and a 26-year-old female from the same family (27), as well as a 55-year-old man.

### Differential expression analysis

Differentially expressed genes (DEGs) between patients with AD or sleep disorder and controls were identified in GSE5281 and GSE40562 datasets using the “limma” package in R (28). DEGs were defined as genes showing adjusted *P* < 0.05. The intersecting DEGs common to the two datasets were identified using the ggVennDiagram package (29), with selection criteria defined as *P* < 0.05 and log_2_(fold change) > 0.5. Only intersecting DEGs were analyzed further.

### Enrichment analysis

We analyzed the intersecting DEGs for enrichment in Gene Ontology (GO) terms and Kyoto Encyclopedia of Genes and Genomes (KEGG) pathways. GO terms, consisting of molecular functions, biological processes, and cell components, were analyzed using the clusterProfiler package (30) in R. *P* < 0.05 was considered to indicate a statistically significant difference.

Fast gene set enrichment analysis (fGSEA) was performed to obtain common pathways using the expression profiles of GSE5281 and GSE40562. Gene set variation analysis (GSVA) is a non-parametric unsupervised analysis method to evaluate the gene set enrichment of chips and transcriptomes (31). The GSVA algorithm was applied to quantify pathways common to AD and sleep disorder. The Cytoscape tool (32) was used to explore interactions among common pathways and genes.

### Co-expression network

We examined the intersecting DEGs using multiscale embedded gene co-expression network analysis (MEGENA), which can effectively improve performance over well-established clustering method. We constructed and analyzed large-scale planar filtered co-expression networks using the MEGENA package in R (33). Filtered networks were used to calculate correlations among intersecting DEGs and thereby identify module genes.

### Protein-protein interaction network construction

PPIs help predict how cells function in health and disease (34). We identified module genes that also participated in pathways involved in both AD and sleep disorder, then we used these genes to construct a PPI network, which was visualized using Cytoscape (32). Among them, the top 10 genes with highest degree of connectivity were identified as hub genes in AD and sleep disorder. Furthermore, common genes to both disorders were used to explore potential functional aspects using Metascape tool (35). The expression of common genes was visualized in a heat map created using “pheatmap” package (23) and compared with the expression of hub genes, which were visualized in AD, sleep disorder, and controls.

### Calculation of immune cell infiltration

The levels of 24 types of immune cells that infiltrated tissues of AD patients, sleep disorder patients and controls were investigated using single-sample gene set enrichment analysis (ssGSEA) and CIBERSORT.^2^ We also used the top 10 genes with highest degree of connectivity in the PPI network and immune cell infiltration data to perform Pearson correlation analysis using the “ggstatsplot” package.

## Results

### Potential functions of differentially expressed genes in Alzheimer’s disease and sleep disorder

**FIGURE 2:**
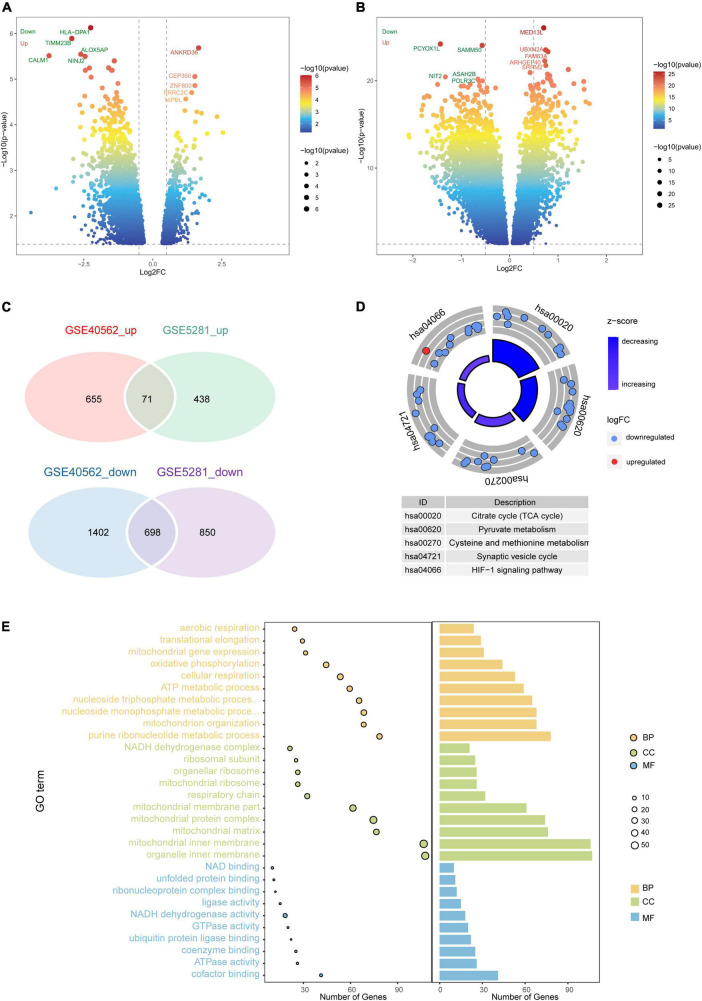
Differential gene expression and enrichment analysis. Volcano plot of differentially expressed genes (DEGs) between **(A)** patients with Alzheimer’s disease (AD) and controls in GSE40562, or **(B)** patients with sleep disorder and controls in GSE5281. Red color indicates upregulated genes; green, downregulated genes. **(C)** Venn diagrams showing common up- or downregulated genes in AD, sleep disorder, and controls. **(D)** Intersecting DEGs were predicted to be involved in five pathways. **(E)** Protein functional analysis in terms of biological processes, cellular components, and molecular functions. GO, Gene Ontology; BP, biological process; CC, cellular component; MF, molecular function; log_2_FC, log_2_(fold change).

A total of 2,057 DEGs were identified in AD patients compared to controls in GSE5281, including 509 upregulated and 1,548 downregulated genes (Figure 2A). We identified 2,826 DEGs between sleep disorder patients and control individuals in GSE40562, including 726 upregulated and 2,100 downregulated genes (Figure 2B).

Among the DEGs, 769 were intersecting DEGs found in both GSE5281 and GSE40562 (71 upregulated and 698 downregulated) (Figure 2C). Intersecting DEGs were involved mainly in the hypoxia-inducible family (HIF)-1 signaling pathway and tricarboxylic acid (TCA) cycle (Figure 2D). The intersecting DEGs were enriched in the GO biological processes of cellular respiration and adenosine triphosphate metabolism, the GO cell components of mitochondrial inner membrane and mitochondrial protein complexes, and the GO molecular functions of nicotinamide adenine dinucleotide (NAD) binding and NADH dehydrogenase activity (Figure 2E).

**FIGURE 3:**
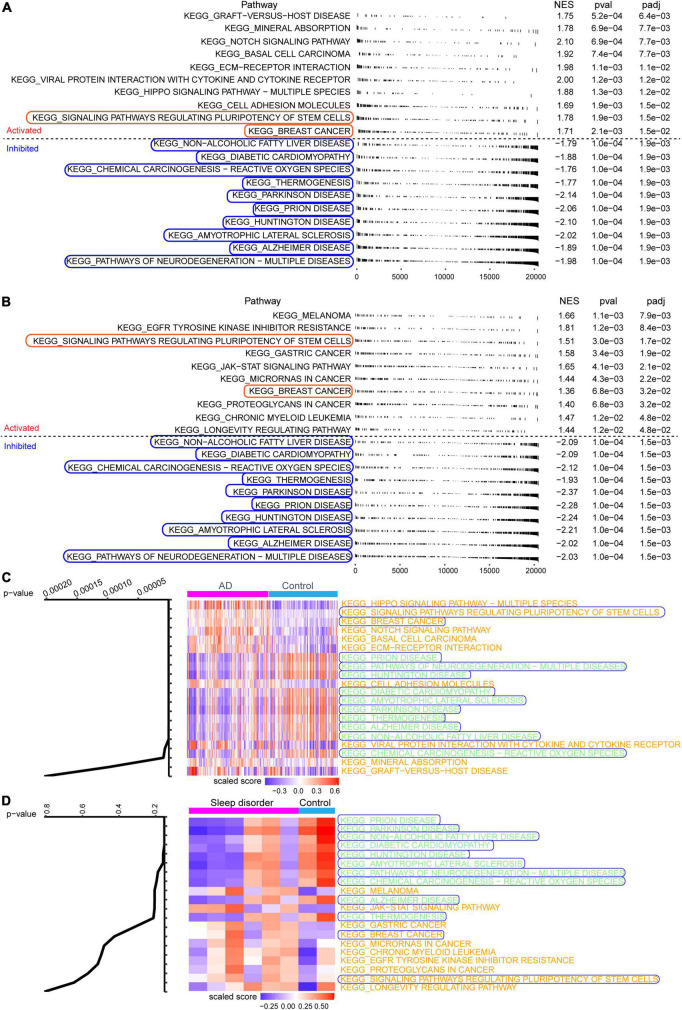
Fast gene set enrichment analysis (fGSEA) and gene set variation analysis. Top 10 pathways significantly activated (red) or inhibited (blue) in the **(A)** GSE5281 or **(B)** GSE40562 dataset, based on fGSEA. **(C,D)** Heat map showing the Kyoto Encyclopedia of Genes and Genomes (KEGG) enrichment analysis in Alzheimer’s disease (AD), sleep disorder (fatal familial insomnia), and controls.

fGSEA showed that some intersecting DEGs were involved in the activation of signaling pathways regulating the pluripotency of stem cells and breast cancer, while others were linked to inhibition of AD and Parkinson’s disease (Figures 3A,B). Our results identified 12 pathways common to AD and sleep disorder: non-alcoholic fatty liver disease, diabetic cardiomyopathy, chemical carcinogenesis-reactive oxygen species, thermogenesis, Parkinson’s disease, prion disease, Huntington’s disease, amyotrophic lateral sclerosis, AD, and pathways related to neurodegeneration in multiple diseases. Twenty pathways were quantified by GSVA, which identified 12 pathways with high scores common to AD and sleep disorder (Figures 3C,D).

### Co-expression network analysis to identify modules and module genes

**FIGURE 4:**
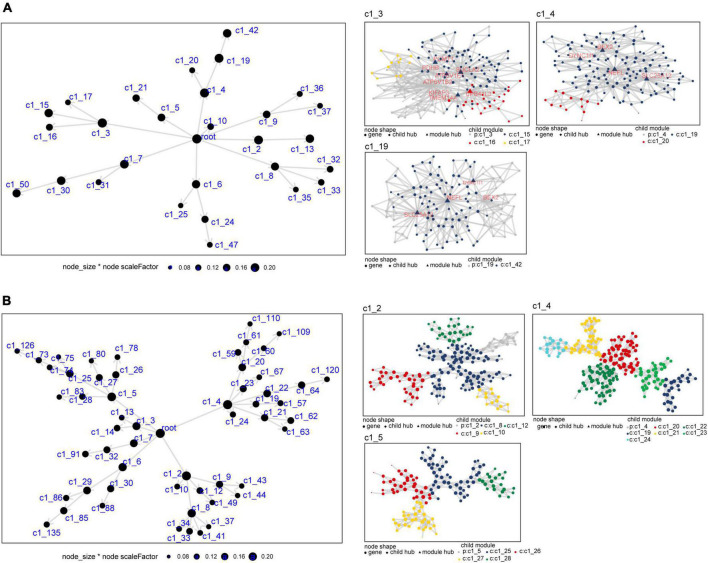
Multiscale embedded gene co-expression network analysis of common genes in Alzheimer’s disease (AD) and sleep disorder. **(A)** Differentially expressed genes in the co-expression network for the GSE5281 dataset. The three largest gene modules are shown: C1_3, C1_4, and C1_19. **(B)** Differentially expressed genes in the co-expression network for the GSE40562 dataset. The three largest gene modules are shown: C1_2, C1_4, and C1_5. Each node represents a module, with the larger nodes indicating a higher number of genes. Each color represents one module, and triangles represent key genes in the module.

We constructed a MEGENA network using the common genes to obtain module genes in AD and sleep disorder. We identified 29 modules and 1,498 module genes in GSE5281 (Figure 4A). The largest modules (C1_3 and C1_4) consisted of 135 genes, followed by module C1_19 with 125 genes. Moreover, we identified 55 modules and 1,791 module genes in GSE40562. Each of the three largest modules (C1_2, C1_4, and C1_5) had 135 genes (Figure 4B).

### Regulation networks and expression of genes common to Alzheimer’s disease and sleep disorder

To identify potential pathways involved in the mechanisms of sleep disorder and AD, genes of the identified 12 pathways that were also module genes were selected as genes that may promote the development of both AD and sleep disorder.

**FIGURE 5:**
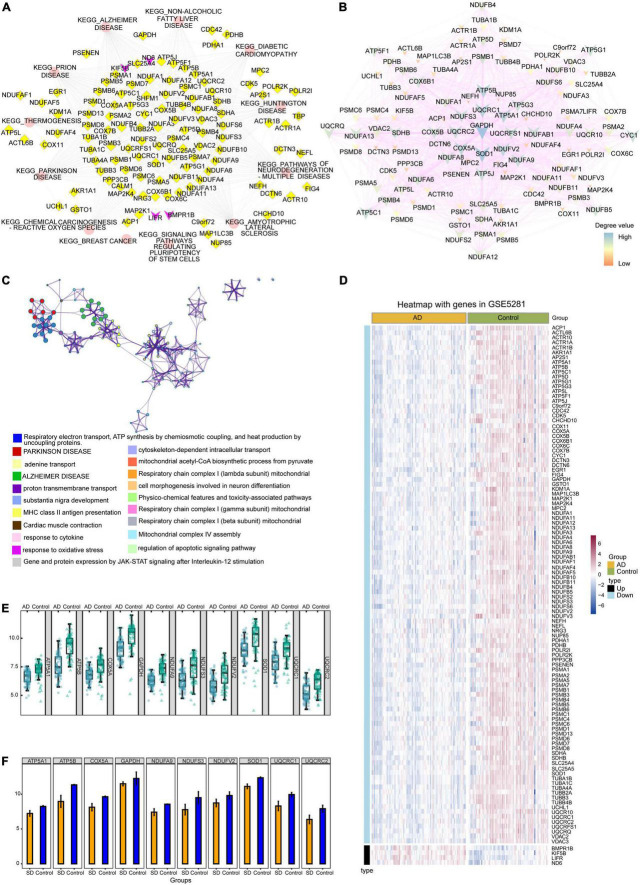
Protein-protein interaction (PPI) network and expression analysis. **(A)** Interaction of the 12 common pathways and common genes analyzed by Cytoscape. Red ellipses indicate pathways; yellow diamonds, downregulated genes; purple V-symbols, upregulated genes. **(B)** Interaction of common genes in the PPI network. Low-degree values are indicated by the small nodes and bright color, while high-degree values are indicated by larger nodes and dark color. **(C)** Functional enrichment analysis showed that common genes were involved mainly in Parkinson’s disease and Alzheimer’s disease (AD). **(D)** Heat map showing the expression of common genes in the GSE5281 dataset. **(E)** Box diagram showing degrees of connectivity of the top 10 genes in AD and controls. **(F)** Bar plot showing the degree values of the top 10 genes in sleep disorder and control group. SD, sleep disorder.

The potential role of gene-regulated pathways was analyzed by Cytoscape (Figure 5A). Similarly, Figure 5B showed that interactions among the genes common to AD and sleep disorder could influence the development of both disorders. Metascape enrichment analysis showed that common genes regulated proton transmembrane transport in sleep disorder and AD (Figure 5C). Heat maps showed the expression of 110 common genes in GSE5281 and GSE40562 datasets, of which four were upregulated and 106 downregulated (Figure 5D and Supplementary Figure 1). Furthermore, the top 10 genes with highest degree of connectivity (ATP5A1, ATP5B, COX5A, GAPDH, NDUFA9, NDUFS3, NDUFV2, SOD1, UQCRC1, and UQCRC2) were identified as hub genes in sleep disorder and AD. Interestingly, 10 hub genes were downregulated in patients with AD or sleep disorder relative to the corresponding controls (Figures 5E,F).

### Immune cell infiltration in Alzheimer’s disease and sleep disorder

**FIGURE 6:**
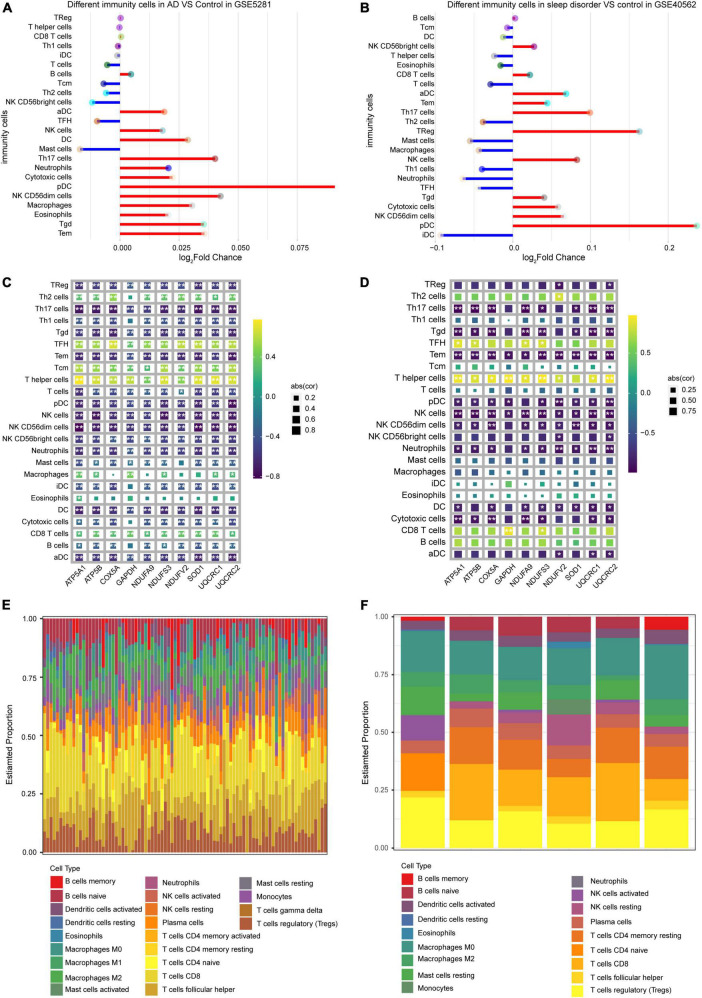
Level of immune cell infiltration in Alzheimer’s disease (AD) and sleep disorder. Comparison of immune cell infiltration **(A)** between AD patients and controls, and **(B)** between sleep disorder patients and controls, based on ssGSEA. Plots of correlations between hub genes and immune cell types in **(C)** AD and **(D)** sleep disorder. Proportions of 22 types of infiltrating immune cells in **(E)** AD and **(F)** sleep disorder, based on the CIBERSORT algorithm.

Infiltration of immune cells was compared between AD or sleep disorder patients and controls in both datasets. Plasmacytoid dendritic cells and T helper 17 (Th17) cells had the most extensive infiltration in both datasets (Figures 6A,B), according to ssGSEA. To further verify the correlation between hub genes and infiltrating immune cells, we performed a Pearson correlation analysis. We found a significant correlation between hub genes and infiltrating immune cells in the GSE5281 (Figure 6C) and GSE40562 datasets (Figure 6D). Moreover, in our CIBERSORT analysis, we found that CD8 + T cells constituted the highest proportion of infiltrated immune cells in GSE5281 (Figure 6E) and GSE40562 (Figure 6F), suggesting that these cells may play a role in the development of AD and sleep disorder.

## Discussion

AD is frequently characterized by disturbed or absent sleep, similarly to sleep disorders. In addition, sleep disturbance is a risk factor for neurodegenerative diseases that are ultimately fatal. However, few studies have looked in detail at molecular pathways that may be common to both disorders. In our study, we integrated transcriptomic data from patients with either condition and controls and performed bioinformatics analyses to identify common pathogenic mechanisms.

Enrichment analysis showed that intersecting DEGs in GSE5281 and GSE40562 were involved in the HIF-1 signaling pathway and the TCA cycle, which produces energy for the cell. HIF appears to play a role in brain response to hypoxia and has been associated with the development of AD (36) and Parkinson’s disease (37). In neurodegenerative diseases, HIF signaling may be associated with impaired brain response to hypoxia. Furthermore, during the development and occurrence of AD and other neurodegenerative disorders, the TCA cycle in mitochondria may be altered. Enzymes and metabolites involved in these reactions suffer various changes during neurodegeneration (38). Patients with sleep disorder show abnormalities in metabolic pathways involving glucose and amino acids, and these pathways interact with the TCA cycle (39, 40).

The results of our GSEA showed that DEGs common to AD and sleep disorder were associated with activation of signaling pathways regulating pluripotency of stem cells and breast cancer, as well as associated with inhibition of Alzheimer’s disease, Parkinson’s disease, and other pathways. Pluripotent stem cells enable the acquisition of a large number of neural cells to improve cell recovery after neurodegenerative disorders, such as AD or Parkinson’s disease (41). Furthermore, women with breast cancer can also have cognitive problems (42). Interestingly, there is a possible connection between sleep disorder and breast cancer (43).

We identified several hub genes common to sleep disorder and AD: ATP5A1, ATP5B, COX5A, GAPDH, NDUFA9, NDUFS3, NDUFV2, SOD1, UQCRC1, and UQCRC2. ATP5A1 has been identified as the target of some components of *Lavandula angustifolia* extract, suggesting that some extract components may have therapeutic effects on AD and other neurodegenerative diseases (44). Downregulated ATP5B was also previously identified as a hub gene involved in AD (45). COX5A plays a vital role in aging-related memory impairment (46). Overexpression of glyceraldehyde-3-phosphate dehydrogenase in AD animal models increases apoptosis of hippocampal cells, neural degeneration, and cognitive dysfunction (47). NDUFA9, NDUFV2, and NDUFS3 are considered critical genes in oxidative phosphorylation and Parkinson’s disease, Huntington’s disease, and AD pathways (48). SOD1 (49) and UQCRC2 (50) have been linked to neurodegeneration. ATP5B (51), GAPDH (52), and SOD1 (53) were reported as sleep deprivation and sleep disorders related genes. One study showed an association among DNA methylation, UQCRC1 expression and risk of AD in a Chinese population (54). Notably, these genes may be potential markers or key genes in AD or other neurological diseases, but they have rarely been investigated in sleep disorders.

In our ssGSEA analysis, plasmacytoid dendritic cells and Th17 cells showed the most extensive infiltration in both AD and sleep disorder patients. Plasmacytoid dendritic cells in the central nervous system have been identified as the primary infiltrating cells in experimental autoimmune encephalomyelitis (55). Additionally, Th17 cells have been associated with cognitive impairment in multiple sclerosis and AD (56). Th17 cells strongly contribute to chronic neuroinflammation, thus perpetuating neurodegenerative processes (57). However, Th17 cells also have a role in barrier protection in many inflammatory diseases (58). Notably, when we applied the CIBERSORT algorithm, the abundance of CD8 + T cell infiltration was the highest in both AD and sleep disorder patients. A previous study found increased numbers of CD8 + T cells in the cerebrospinal fluid in AD patients (59). Differences in the results of the two immune infiltration analyses may be due to differences among individual patients.

This study presents several limitations. The main limitation is that our results are based purely on bioinformatics of small samples. Our analyses should be validated and extended in experimental studies. Furthermore, the infiltration of different immune cells may vary among patients, so we need to validate our results in large samples.

## Conclusion

In the present study, ATP5A1, ATP5B, COX5A, GAPDH, NDUFA9, NDUFS3, NDUFV2, SOD1, UQCRC1, and UQCRC2 were identified as hub genes differentially expressed in AD and sleep disorder relative to healthy individuals. These genes may be involved in AD and sleep disorder through pathways related to neurodegeneration and multiple diseases, and they may be candidate biomarkers to facilitate diagnosis and therapy. We also identified some immune cell populations that strongly infiltrate tissues in both disorders and which therefore may contribute to pathogenesis.

## Data availability statement

The datasets presented in this study can be found in online repositories. The names of the repository/repositories and accession number(s) can be found in the article/Supplementary material.

## Ethics statement

The studies involving human participants were reviewed and approved by the First People’s Hospital of Nanning. Written informed consent for participation was not required for this study in accordance with the national legislation and the institutional requirements. Written informed consent was obtained from the individual(s) for the publication of any potentially identifiable images or data included in this article.

## Author contributions

LL, JY, XH, DZ, and YL conceived and designed the study. All authors analyzed the data, prepared the figures and tables, wrote the manuscript, reviewed, the manuscript and approved its submission.

## Footnotes

1
https://www.ncbi.nlm.nih.gov/geo/

2
https://cibersort.stanford.edu/

## Supplementary material

Supplementary Figure 1 Heat map showing the expression of common genes in the GSE40562 dataset.
