# Supplementary figures and images for: Identification of molecular signatures associated with sleep disorder and Alzheimer’s disease
Source: Front Psychiatry. 2022 Aug 4;13:925012. doi: 10.3389/fpsyt.2022.925012 (PMC9386361; doi:10.3389/fpsyt.2022.925012)

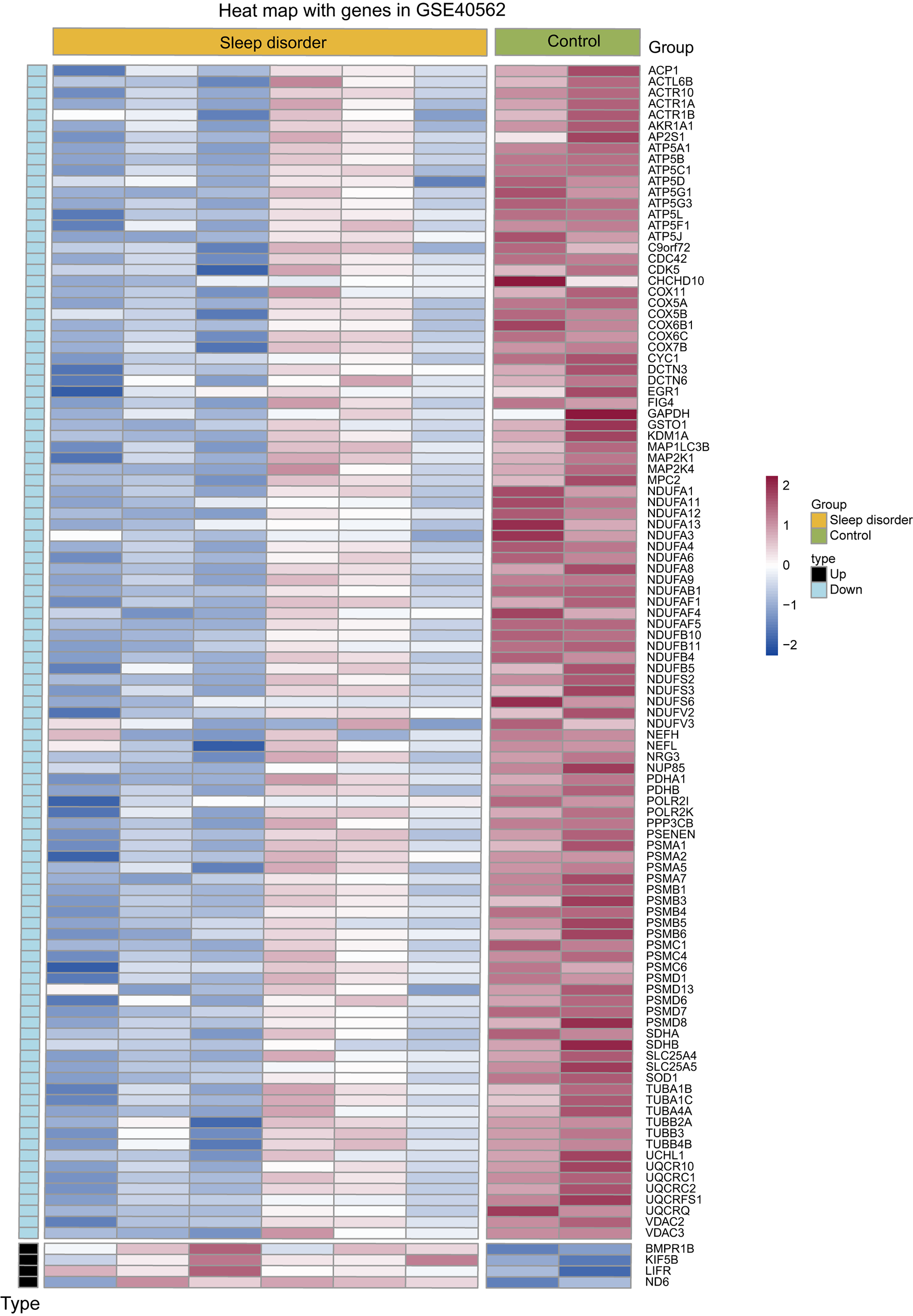

Supplement: Supplementary Figure 1 — Heat map showing the expression of common genes in the GSE40562 dataset. [file Image_1.TIF]
